# Virus Host Protein Interaction Network Analysis Reveals That the HEV ORF3 Protein May Interrupt the Blood Coagulation Process

**DOI:** 10.1371/journal.pone.0056320

**Published:** 2013-02-13

**Authors:** Yansheng Geng, Jun Yang, Weijin Huang, Tim J. Harrison, Yan Zhou, Zhiheng Wen, Youchun Wang

**Affiliations:** 1 Department of Cell Biology, National Institutes for Food and Drug Control, No 2 Tian Tan Xi Li, Beijing, China; 2 Health Science Center, Hebei University, Baoding, China; 3 Department of Surgery, St Jude Children's Research Hospital, Memphis, Tennessee, United States of America; 4 Division of Medicine, University College London Medical School, London, United Kingdom; Drexel University College of Medicine, United States of America

## Abstract

Hepatitis E virus (HEV) is endemic worldwide and a major cause of acute liver disease in developing countries. However, the molecular mechanisms of liver pathology and clinical disease are not well understood for HEV infection. Open reading frame 3 (ORF3) of HEV encodes a small phosphoprotein, which is assumed to be involved in liver pathology and clinical disease. In this study, the interactions between the HEV ORF3 protein and human proteins were investigated using a stringent, high-throughput yeast two-hybrid (Y2H) analysis. Thirty two proteins were shown to interact with genotype 1 ORF3, 28 of which have not been reported previously. These novel interactions were evaluated by coimmunoprecipitation of protein complexes from transfected cells. We found also that the ORF3 proteins of genotype 4 and rabbit HEV interacted with all of the human proteins identified by the genotype 1 ORF3 protein. However, the putative ORF3 protein derived from avian HEV did not interact with the majority of these human proteins. The identified proteins were used to infer an overall interaction map linking the ORF3 protein with components of the host cellular networks. Analysis of this interaction map, based on functional annotation with the Gene Ontology features and KEGG pathways, revealed an enrichment of host proteins involved in complement coagulation, cellular iron ion homeostasis and oxidative stress. Additional canonical pathway analysis highlighted the enriched biological pathways relevant to blood coagulation and hemostasis. Consideration of the clinical manifestations of hepatitis E reported previously and the results of biological analysis from this study suggests that the ORF3 protein is likely to lead to an imbalance of coagulation and fibrinolysis by interacting with host proteins and triggering the corresponding pathological processes. These results suggest critical approaches to further study of the pathogenesis of the HEV ORF3 protein.

## Introduction

Hepatitis E, caused by hepatitis E virus (HEV), is an important public health problem in many developing countries. The disease usually is acute and self-limited with typical symptoms of jaundice, dark urine, anorexia, enlarged tender liver, elevated ALT levels and abdominal pain and tenderness, accompanied by nausea, vomiting and fever [1], [2]. However, acute hepatitis E can also progress to fulminant hepatitis with encephalopathy and coagulation disorders. In these cases, the patient's prothrombin index and accelerin levels were lower and death was more frequent [3], [4]. In epidemics, a relatively high mortality of up to 20–30% has been reported in infected pregnant women [5], with the characteristics of a short pre-encephalopathy period, rapid development of cerebral oedema and a high rate of occurrence of disseminated intravascular coagulation that may represent a severe manifestation of Schwartzmann-like phenomenon [6]. It has been reported recently that HEV infection in immunocompromised patients can evolve to chronic hepatitis that progresses rapidly to cirrhosis [7]. Therefore, disturbances of the coagulation/fibrinolysis system are common and linked to poor prognosis in hepatitis E patients. Immune-mediated injury of liver cells has been postulated to be the primary cause of HEV associated diseases [2]. Nevertheless, the molecular mechanism responsible for liver pathology and clinical disease in hepatitis E is not well understood.

Hepatitis E is known to be a zoonotic disease. In addition to humans, HEV has been detected in a variety of animals [8]. Based on nucleotide sequence comparisons, the known isolates of mammalian HEV can be divided into four genotypes, numbered 1 to 4 [9], [10]. Genotypes 1 and 2 have been responsible for large epidemics in the human population, transmitted mostly by the fecal-oral route through contaminated water, are endemic throughout Asia and Africa and have been isolated only from humans. Genotype 3 HEV has been isolated worldwide from humans and animals including pigs, wild boar, deer and mongoose and is responsible for the most sporadic cases of hepatitis E in developed countries. Genotype 4 is spread mainly in Asian countries and isolated from sporadic human cases and domestic pigs [2], [8], [11]. Avian HEV was firstly isolated in the United States and was found to be widespread in countries of Europe, Australia and China [12], [13], [14]. Avian HEV infects chickens and turkeys but not pigs or monkeys [8]. More recently, a novel HEV, designated rabbit HEV, was found to be prevalent in farmed rabbits in China, but less so in pigs [15], [16]. The reason for the differences in host range among the various genotypes is unknown. Thus, extensive comparative analyses are required to determine which variables at the molecular level are relevant to host tropism.

HEV belongs to the genus Hepevirus of the family *Hepeviridae* [17]. It is a non-enveloped virus, approximately 27–34 nm in size and contains a single stranded, positive sense RNA of approximately 7.2∼7.3 kb which encodes three open reading frames (ORFs 1, 2 and 3) [18]. ORF1 encodes a non-structural polyprotein essential for virus replication. ORF2 encodes the viral capsid protein, the major structural protein of the virion. ORF3 encodes a tiny phosphoprotein of 113 or 114 amino acids [19], [20]. It has been reported that the ORF3 protein interacts not only with the ORF2 protein [21] but also with several cellular proteins, including microtubules in the cytoskeleton [22], [23], a1-microglobulin/bikunin precursor, bikunin [24], hemopexin [25], tumor susceptibility gene 101 (Tsg101) and the src homology 3 domains [26], [27], [28]. It may act to modulate the acute-phase disease response [29], protect cells from mitochondrial depolarization [30] and enhance the expression of glycolytic pathway enzymes [31]. Moreover, the ORF3 protein may be involved in virus egress or release from infected cells [32]. However, the precise function of ORF3 protein at a global level is far from clear.

A continued effort to define the interactions between virus proteins and host proteins may provide a better understanding of how viruses replicate and cause disease, and will enable comparisons of the machineries that different genotype viruses use to manipulate host cells. In an attempt to understand more completely the function of the HEV ORF3 protein in virus infection, replication and pathogenesis, in this study we used the yeast two-hybrid assay to screen the ORF3 gene against a human liver cDNA library. The human proteins found to interact with the genotype 1 HEV ORF3 protein were validated and their capacity to bind the genotype 4, rabbit HEV and avian HEV ORF3 proteins also were evaluated. Based on the Y2H screen results, the first interaction network between HEV ORF3 and host proteins was created and the potential biological roles of these proteins were analyzed in this study.

## Results and Discussion

### HEV ORF3 and human protein interactions identified by yeast two-hybrid analysis

To identify host factors that may participate in HEV infection and the pathogenic process, we sought to identify human proteins that are directly affected by physical associations with HEV ORF3 protein. We used genotype 1 HEV ORF3 as bait to screen a human liver cDNA library through yeast two-hybrid mating. The interaction of each pair of bait and prey identified by mating was confirmed by co-transformation of yeast. Three independent screenings were conducted to avoid leakiness of positive interactions. After elimination of autoactivators, 32 human proteins interacting with the HEV ORF3 protein were identified (Table 1). Of these 32 proteins, 6 (19%) were identified in all three screenings, 7 (22%) were identified in two and the other 19 (59%) were identified only in one screening. Twenty eight of the 32 interactions were novel findings in this study while the other four proteins, including FGB, AMBP, TSG101 and HPX, have been reported previously [24], [25], [27], [33] (Table 1). However, two host cellular proteins, microtubles and CIN85, which have been reported to interact with the ORF3 protein [23], [34] were not identified in this study.

**Table 1:** Human liver proteins interacting with the HEV ORF3 protein.

GENE ID	Official Symbol	Description
350	APOH	Apolipoprotein H (beta-2-glycoprotein I)
3240	HP	Haptoglobin
2512	FTL	Ferritin, light polypeptide
3263	*HPX	Hemopexin, a heme-binding protein that transports heme to the liver
967	CD63	CD63 molecule
7018	TF	Transferrin
325	APCS	Amyloid P component
2244	*FGB	Fibrinogen beta chain
259	*AMBP	Alpha-1-microglobulin/bikunin precursor
7448	VTN	Vitronectin
1571	CYP2E1	Cytochrome P450, family 2, subfamily E
4513	COX2	Cytochrome c oxidase subunit II
10287	RGS19	Regulator of G-protein signaling
4143	MAT1A	Methionine adenosyltransferase I, alpha (MAT1A)
9	NAT1	N-acetyltransferase1 (arylamine N-acetyltransferase)
462	SERPINC1	Serine proteinase inhibitor, clade C, member 1
229	ALDOB	Aldolase B, fructose-bisphosphate
2395	FXN	Homo sapiens frataxin (FXN), mitochondrial
3312	HSPA8	Heat shock 70 kDa protein 8
1364	DCN	Decorin
10296	MAEA	Homo sapiens macrophage erythroblast attacher
3478	IGFBP4	Insulin-like growth factor binding protein 4
710	SERPING1	Serine proteinase inhibitor, clade C, member 1
4502	MT2A	Homo sapiens metallothionein 2A
95	ACY1	Aminoacylase 1
8431	NR0B2	Nuclear receptor subfamily 0, group B, member 2
100144686	CD151	CD151 molecule (Raph blood group)
3081	HGD	Homogentisate 1,2-dioxygenase (homogentisate oxidase)
3627	CXCL10	CXCL10 chemokine (C-X-C motif) ligand 10
4728	NDUFS2	NADH dehydrogenase (ubiquinone) Fe-S protein
7251	*TSG101	Homo sapiens tumor susceptibility gene 101 protein
2243	FGA	Fibrinogen alpha chain
30011	†SH3KBP1	SH3-domain kinase binding protein 1, CIN85

* Proteins interacting with ORF3 published previously and identified in this study;

† Protein interacting with ORF3 published previously but not identified in this study.

Yeast two-hybrid screening has proven to be highly versatile in its applicability to a wide range of proteins. However, false positives frequently are observed [35]. In the Y2HGold system, protein-protein interactions are identified on the basis of activation of four separate reporter genes AUR1-C, HIS3, ADE2, and MEL1 in yeast cell, as indicated by growth of the mated yeast colonies on SD/-Ade/-His/-trp/-leu/X-α-Gal/AbA medium. The AUR1-C gene encodes the enzyme inositol phosphoryl ceramide synthase and the expression this enzyme confers strong resistance to the otherwise highly toxic drug, Aureobasidin A (AbA). MEL-1 encodes a-galactosidase, an enzyme occurring naturally in many yeast strains, yeast colonies that express Mel1 turn blue in the presence of the chromagenic substrate X-a-Gal. Y2HGold is unable to synthesize histidine and adenine and therefore unable to grow on media that lack these essential amino acids. When bait and prey proteins interact, Gal4-responsive His3 and ADE2 permit the cell to synthesize these two amino acids and grow on His and Ade minimal medium. Because all of the four independent reporter genes are used selection of protein-protein interactions in this system, the occurrence of false interactions is limited. Repetition and confirmation of the screening experiment allowed us to be very stringent in obtaining a high quality set of 32 human proteins that interacted with the HEV ORF3 protein.

### Comparison of interactions between different genotype ORF3 proteins and host proteins

The ORF3 proteins of genotypes 1, 2 and 4 comprise 114 amino acids while the ORF3 proteins of genotype 3 and rabbit HEV comprise 113 amino acids [15], [19], [20]. Compared with ORF2 from different HEV genotypes, ORF3 is more variable. The amino acid sequence identities of ORF3 among genotypes 1 to 4 and rabbit HEV are 78–89% [15]. Genotype-specific regions and sites can be found in the ORF3 protein when the amino acid sequences of representative strains of various genotypes are aligned [36].

In order to determine whether the interactions of the ORF3 protein with host proteins varies among different genotypes, we cloned the ORF3 genes of genotype 4, rabbit HEV and avian HEV into pGBKT7. Each ORF3 constructin pGBKT7 was co-transformed into yeast Y2HGold with each of the 32 prey constructs initially identified using the ORF3 of genotype 1. Intriguingly, we found that the genotype 4 and rabbit HEV ORF3 proteins interacted with all of the 32 human proteins. Thus, no differences were found among the ORF3 proteins of genotype 1, 4 and rabbit HEV in their interactions with human proteins. These results indicate that the mechanisms used to interact with host cells are relatively conserved among different mammalian HEV genotypes.

Avian HEV has been suggested to constitute a new genus in the family *Hepeviridae* [8]. The putative avian HEV ORF3 protein is comprised of only 87 amino acids, with 29–34% identity to the mammalian HEV ORF3 [37]. Using avian HEV ORF3 as bait to cotransform yeast Y2HGold with each of the 32 prey vectors, 13 (40%) positive interactions were found. These 13 proteins are AMBP, FTL, COX2, IGFBP4, HSPA8, HGD, ACY1, MAEA, MAT1A1, HP, RGS19, TSG101, and MT2A. Although various domains that seem to interact with host proteins are conserved in the avian ORF3 protein, 60% of the human proteins identified above did not interact with the avian ORF3 protein. This may be attributable to divergence of the human proteins from their avian equivalents, divergence of the functions of mammalian and avian ORF3 proteins or a combination of these possibilities.

### Validation of the interactions

To evaluate the screening results from the yeast hybrid system we sought to verify the ORF3 and human protein interactions identified by co-immunoprecipitation of the binding partners from transfected mammalian cells. In this assay, the HEV ORF3 cDNA was cloned into the pAcGFP1-C vector to express fusion proteins containing an AcGFP1 tag. This tag functions as the epitope for immunoprecipitation. Each of the cDNAs of the human proteins identified above was cloned into the pProLabel-C vector to incorporate the ProLabel tag, which enables chemiluminescent detection. The pair of vectors expressing ORF3-GFP1 and prolable-prey fusion proteins was cotransfected into 293 cells. Total protein was harvested 48 h after transfection and protein complexes were precipitated with polyclonal antibody against ORF3 protein. Precipitated proteins were tested for the ability of the prolable tag to activate its substrate and generate a chemiluminescent signal. Because the interaction between the ORF3 protein and HPX had been shown previously by co-immunoprecipitation [25], this pair was used as positive control for validation of the other interactions.

**Figure 1:**
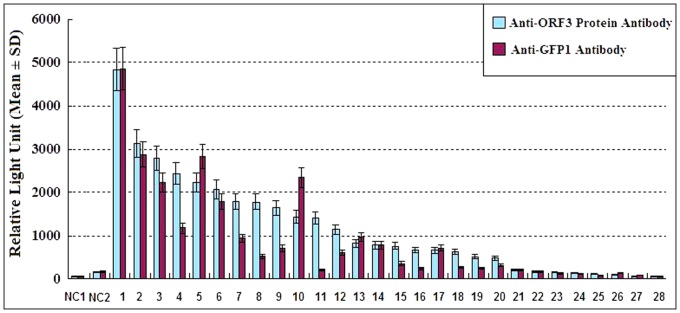
Chemiluminescent detection of protein-protein interactions by co-immuno precipitation. On the x-axis, “NC1 and NC2” represent 293 cell lysate and negative control; “1∼28” represent FTL, HP, APCS, APOH, NR0B2, AMBP, VTN, TF, MT2A, COX2, CD151, HPX, IGFBP4, FXN, FGA, ALDOB, HSPA8, CYP2E1, SERPINC1, CD63, RGS19, FGB, NAT1, DCN, TSG101,CXCL10, HGD and MAT1A. In the co-immunoprecipitation, the interacting protein complexes were precipitated by both Anti-HEV ORF3 polyclonal antibody(indicated by light blue squares) and GFP1 polyclonal antibody (indicated by magenta squares)., The negative controls were precipitated by Anti-AcGFP antibody.

The chemiluminescent intensity of the negative control (the pair of GFP1-lam and Prolable-T) was 157±63RLU and that of positive control, HPX, was 1140±178 RLU. The signal intensity of 11 proteins FTL, HP, APCS, APOH, NR0B2, AMBP, VTN, TF, MT2A, COX2, CD151, appeared higher than HPX (Fig. 1). Thus, at least these 12 proteins including HPX also were shown to interact with HEV ORF3 by co-immunoprecipitation from mammalian cells. In addition, the IGFBP4, FXN, FGA, ALDOB, HSPA8, CYP2E1, SERPINC1, CD63-, TF-, FGB-, FGA-, CYP2E1-ORF3 pairs yielded significantly higher levels of prolable activity than the negative control (Fig. 1). The interaction of FGB with ORF3 has been shown previously by multiple methods but has not been confirmed by co-immunoprecipitation. We consider this set to represent positives in this assay. Co-immunoprecipitation of the prolable-tag and ORF3 protein, as well as GFP1 and each of the 28 preyed proteins were also conducted. No obvious difference was found by comparing the chemiluminescent intensity of the negative control and those of the protein pairs. This result indicates that there is no interaction between the prolable-tag and ORF3 protein and between GFP1 and each of the tested liver proteins.

To confirm the results, another Co-IP experiment with polyclonal antibody against GFP1 had been done. The result was generally in accordance with that by using anti-ORF3 antibody, but RLU values for most of the protein pairs were little lower (Fig. 1). The chemiluminescent intensity of the negative control was 172±59 RLU and that of positive control, HPX, was 614±134 RLU (Fig. 1). The signal intensity of proteins, FTL, HP, NROB2, COX2, IGFBP4, APCS, AMBP, VTN, APOH, FXN, CD63 and MT2A were higher than HPX.

The interaction of TSG101 with ORF3 also has been reported previously. However, the signal intensity of TSG101 in this assay was relatively low. The results of this co-immnuoprecipitation assay were affected by several factors; thus, the interactions of proteins with lower signal intensities should be confirmed using other assays. Because the fusion proteins of SERPING1, NDUSF2, ACY1 and MAEA with the prolable tag did not show chemiluminescent activity, these four pairs were not co-immunoprecipitated. Thus, the rate of confirmation in this study was similar to that observed for other high quality, large-scale yeast two-hybrid screens, which range from 20 to 85% [38].

### Mapping of HEV ORF3 protein-human protein interactions and topological analysis of the network

Thirteen of the 32 interacting proteins (41%) were liver specific, as shown by GSEA analysis. Hasio identified 255 liver specific genes from a total of 12,000 genes expressed in liver cells [39]. Thus, human proteins interacting with ORF3 were highly overrepresented in the liver specific gene set (13/255 versus 32/12,000, p<0.01). This may imply that the HEV ORF3 protein prefers to interact with proteins specifically expressed in the liver. A significant proportion of these proteins were mapped to the extracellular space (GO:0005615; 5 of 32, 16%; p<0.05), including FGB, FGA, APCS, HPX and VTN. According to the GO terms, these proteins are outside the cells proper, usually taken to be outside the plasma membranes, and in regions occupied by fluid. Since the presence of ORF3 protein in the surface of HEV virion in blood has been demonstrated (Takahashi et al, 2008), further studies will be needed to exploring whether ORF3 protein may interact with these proteins in the blood.

**Figure 2:**
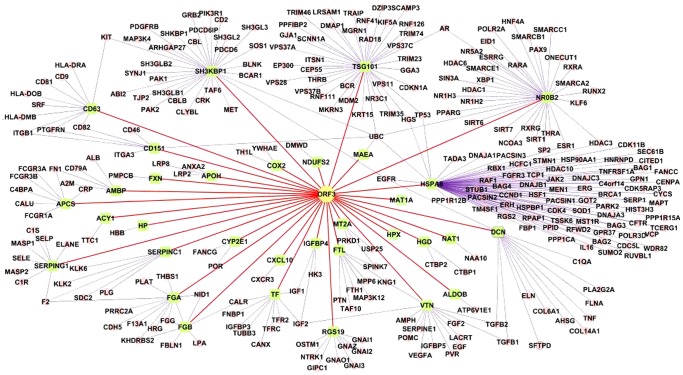
A visualized map of HEV ORF3 protein-human protein interactions. Yellow nodes: HEV ORF3 protein; green nodes: host proteins identified as interacting partners of the HEV ORF3 protein using the Y2HGold system; tiny nodes: secondary interactors of the host proteins interacting with the ORF3 protein.

We mapped the ORF3 protein with the 32 interacting human proteins identified in this study and one protein, CIN85, found from literature into a visualized network (Fig. 2). However, proteins which have been shown to interact with ORF3 but not as a single molecule, such as in microtubles [23] are not included in this network. In the map, 271 secondary interacting human proteins, which interact with the 33 proteins, were generated by searching the linked BioGrid database (Fig. 2). To describe further the topological properties of the HEV interaction network in relation to the entire human interactome, we used Cytoscape to calculate the local (degree) and global (betweenness) centrality measures of the human proteins targeted by ORF3 proteins integrated into the human interactome. Our analysis revealed that the average degree of the network is 8.7, which is significantly higher than the average degree of proteins picked randomly from the human interactome (5.9) [40] and indicates that the ORF3 protein tends to be highly connected or a hub protein in the human interactome.

### Functional analysis of the HEV ORF3–human interaction network

**Figure 3:**
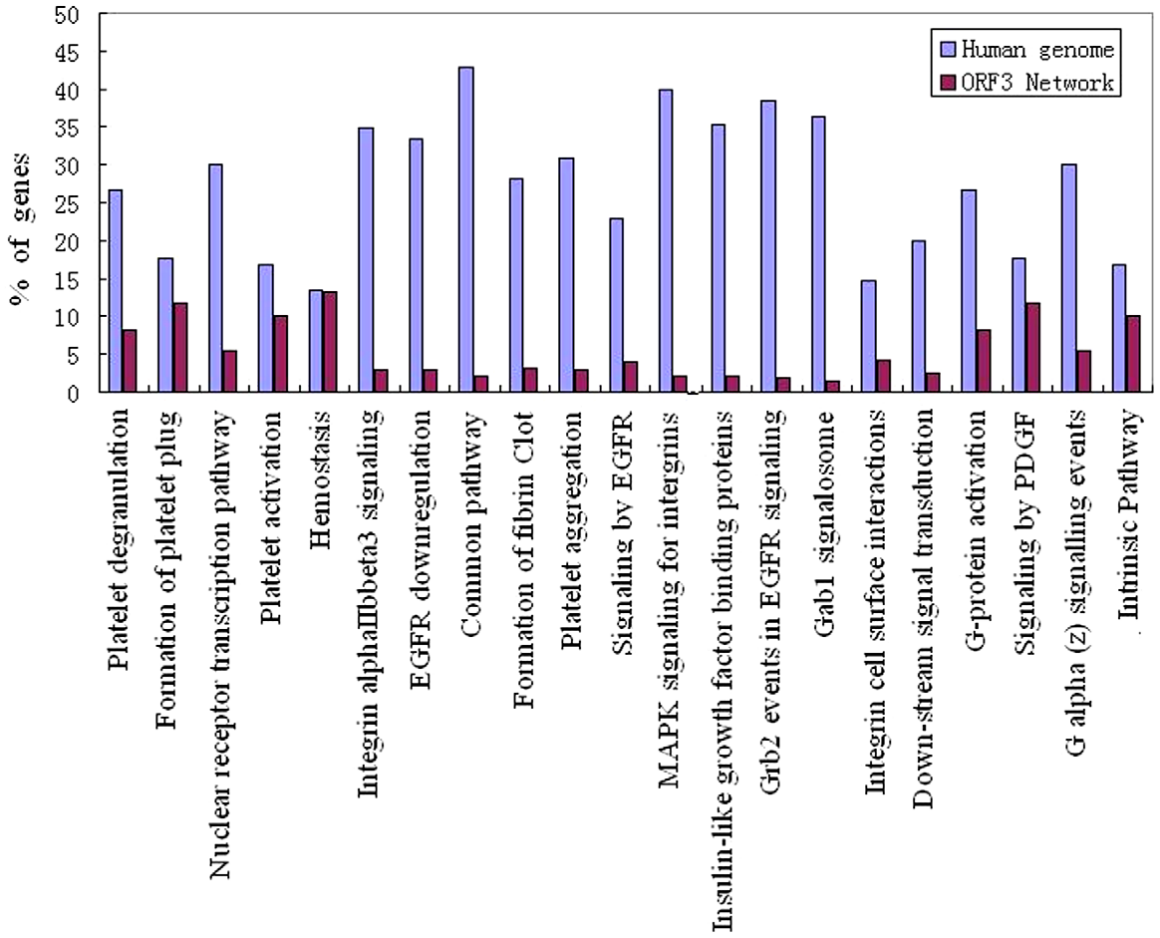
Enriched canonical pathways in the HEV ORF3 protein and human protein interaction network (p≦0.05). The canonical pathways are mapped to the x-axisand the y-axis represents the % of genes mapped to a given pathway within the network and in human genome.

To understand better the biological functions targeted by the HEV ORF3 protein, we tested the enrichment of Canonical pathways for the 33 interacting proteins by analyzing the proteins with regard to the KEGG, Biocart and Reactome functional annotation pathways (Fig. 3, Table 2). We also performed an enrichment analysis of the 33 proteins using the Gene Ontology (GO) database in order to characterize the cellular functions significantly over-represented in the pool of proteins interacting with HEV ORF3 (Table 3).

**Table 2:** Proteins mapped to primary canonical pathways enriched in the HEV ORF3 and human protein interaction network.

Canonical pathways*	Description	Proteins
BIOCART	Prothrombin activation pathway	FGA,FGB,SERPINC1,SERPING1
	Fibrinolysis pathway	FGA,FGB
KEGG	Complement and coagulation cascades	FGA,FGB,SERPINC1,SERPING1
	Oxidative phosphorylation	COX2, NDUFS2
REACTOME	Genes involved in formation of fibrin clot (clotting cascade)	FGA,FGB,SERPINC1,SERPING1
	Genes involved in platelet activation, degranulation and formation of platelet plug	FGA,FGB,SERPING1, TF, CD63
	Genes involved in Grb2:SOS provides linkage to MAPK signaling for intergrins	CYP2E1,NR0B2
	MAPK signaling for intergrins	FGA,FGB
	Integrin cell surface interactions	FGA,FGB,VTN
	Biological oxidations	CYP2E1, NAT1, MAT1A

* Canonical pathways include the Biocart pathway database, KEGG pathway database, Reactome pathway database.

**Table 3:** Gene Ontology (GO) functional enrichment analysis of HEV ORF3 targeted human proteins.

Ontology	Description	GO term	Associated proteins
Molecular function	Serine type endopeptidase inhibitor activity	GO:0004867	AMBP, SERPING1
	Protease inhibitor activity	GO:0004867	AMBP, SERPING1
Cellular component	Extracellular space	GO:0005615	FGB, FGA, APCS, HPX, VTN
Biological process	Cation iron ion homeostasis	GO:0006879	FXN, HPX, FTL,TF,

#### Complement and coagulation

**Figure 4:**
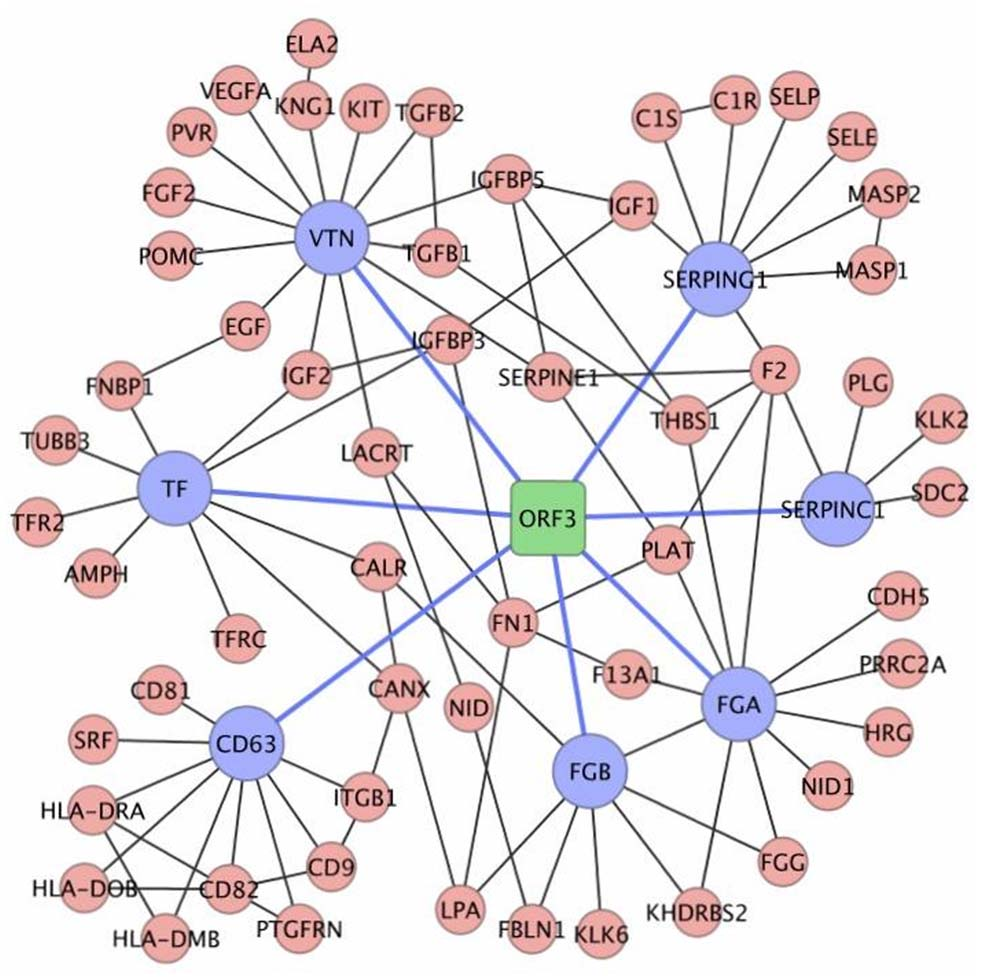
Network illustration of interactions between HEV ORF3 interacting proteins and host proteins associated to “Hemostasis”.

Our Y2H screening identified the fibrinogen alpha chain (FGA), fibrinogen beta chain (FGB) and serine protease inhibitors SERPINC1 and SERPING1 as primary interacting partners of the HEV ORF3 protein (Table 1, Fig. 2). All four proteins are involved in the KEGG pathway “Complement and coagulation cascades” (4 of 32, 12%; Fig. 4), which functions in the host innate immune response against pathogen invasion and clearance of viral antigens from the blood of infected hosts.

Fibrinogen, composed of three pairs of nonidentical polypeptide chains (FGA, FGB and FGG), is a hepatic acute-phase protein and serves as a central molecule that maintains host homeostasis and haemostasis during an acute-phase response. The thrombin cleavage product of fibrinogen, fibrin, has been suggested to play a role as the central regulator of the inflammatory/acute-phase response [41]. The synthesis of FGB is upregulated two to tenfold following infection, tissue injury and inflammation [42], [43]. The interaction of the ORF3 protein with FGB has been reported previously and ORF3 has been thought to be able to attenuate inflammatory responses and create an environment for increased viral replication and survival through downregulating the expression of FGB [33].

SERPING1 is a highly glycosylated plasma protein involved in the regulation of the complement cascade. This protein inhibits activated C1r and C1s of the first complement component and thus regulates complement activation. SERPINC1 is a plasma protease inhibitor and a member of the serpin superfamily. This protein inhibits thrombin, as well as other activated serine proteases of the coagulation system, and regulates the blood coagulation cascade. The ability of viruses to cause disease depends on their capacity to avoid detection and targeting by the host immune response. Perturbing the host immune response via the complement cascade is common strategy for a diverse group of viruses. Interactions with SERPINC1 and SERPING1 may allow the ORF3 protein to perturb the host immune response directly via the complement activation cascade and thus contribute to HEV infection and pathogenesis.

#### Interacting proteins involved in hemostasis

In this study we found that a significant number of proteins in the ORF3 network mapped to the KEGG pathway “Complement and coagulation cascades”. It is understandable that viruses exploit the complement pathway to avoid lysis and at the same time enhance virus uptake, as we discussed above; however, the reason for their involvement with the coagulation pathway is not clear. We used GSEA to carry out an enrichment analysis of the 33 proteins interacting with the ORF3 protein in the canonical pathways. It is noteworthy that FGA, FGB, SERPINC1 and SERPING1 also are involved in multiple canonical pathways associated with hemostasis including the “prothrombin activation pathway’, “formation of fibrin clot”, “platelet activation, degranulation and formation of platelet plug” (Table 2). In addition, the CD63 molecule, transferritin (TF) and vecterin (VTN) also participate in hemostasis. Thus, a significant number (7/32, 22%) of interacting proteins are involved in the blood clotting process (Table 2, Fig. 3).

In the acute phase of hepatitis E, higher levels of alanine aminotransferase and aspartate aminotransferase are associated with severe disease and derangement of coagulation [44]. HEV is a major cause of fulminant hepatitis in endemic regions [4], [45]. Coagulation disorders were important symptoms in cases of fulminant hepatitis Ereported in France, the prothrombin index and accelerin levels were lower and death was more frequent [3]. Women with HEV infection were more likely than those with other forms of viral hepatitis to have antepartum hemorrhage [46]. Fulminant hepatic failure (FHF) caused by HEV in pregnant women also showed a high occurrence of disseminated intravascular coagulation and this may be a severe manifestation of a Schwartzmann-like phenomenon [6], [47]. However, the pathogenesis of coagulation disorders is poorly understood in hepatitis E. Binding to and alteration of the properties of these hemostasis associated proteins by the ORF3 protein may be the molecular feature that links the clinical and pathological relationship between HEV infection and haemostatic abnormalities.

The function of fibrinogen in hemostasis is well established. Following vascular injury, fibrinogen is cleaved by thrombin to form fibrin, which is the most abundant component of blood clots, and the absence of fibrinogen from the plasma leads to prolonged bleeding. In this study, both the α and β chains (FGA and FGB) of fibrinogen are found to interact with the ORF3 protein. The interaction of FGB has been confirmed previously by multiple experiments and it was found that ORF3 downregulates the expression FBG [33]. Therefore, we propose here that the decrease of expression FGB effected by ORF3 may result in a low concentration of fibrinogen in the blood, thus leading to the pathological disorder of hemostasis.

The SERPINC1 and SERPING1 proteins have been shown in this study to interact with the ORF3 protein. SERPINC1 has an important role in the coagulation process. It is found in the bloodstream and is important for controlling blood clotting. SERPINC1 is also called antithrombin and is a serine protease inhibitor that helps control several biochemical processes by blocking the activity of certain proteins. CD63 is a member of the transmembrane-4 superfamily and may function as a blood platelet activation marker. Both CD63 and TF are involved in platelet adhesion and degranulation. Vitronectin (VTN) is a glycoprotein present in plasma, the extracellular matrix and in the α-granules of platelets. VTN participates in the regulation of humoral responses, such as coagulation, fibrinolysis, and activation of the complement cascade. VTN has been detected on the surface of platelets and has been implicated in platelet adhesion and aggregation [48], [49]. The ORF3 protein may affect blood coagulation by targeting multiple proteins involved in hemostasis.

#### Cation homeostasis

We also carried out an enrichment analysis of the 32 proteins using the Gene Ontology (GO) database. Five proteins, ferritin light polypeptide (FTL), hemopexin (HPX), haptoglobin (HP), frataxin (FXN) and metallothionein 2A (MT2A) mapped to biological processes associated with cellular cation homeostasis (Go:0030003). Further analysis showed that 4 of the 5 proteins are involved in cellular iron cation homeostasis (Go: 0006879) (Table 3), maintenance of an internal steady state of iron cations in the cell. Thus, the ORF3 protein may cause an imbalance of iron cation homeostasis by targeting these proteins. However, it is not clear whether disturbances in host iron metabolism cause or result from hepatitis E disease progression.

Haptoglobin functions to bind free plasma hemoglobin, which allows degradative enzymes to gain access to the hemoglobin, while at the same time preventing loss of iron through the kidneys and protecting the kidneys from damage by hemoglobin. HPX transports heme from the plasma to the liver and may be involved in protecting cells from oxidative stress. FXN is required for cellular respiration and has been suggested to regulate mitochondrial iron homeostasis; it is a key activator of mitochondrial energy conversion and oxidative phosphorylation [50]. Evidence has accumulated that iron restriction is an important component of innate immunity [51]. We also note that TF, HP, HPX, as well as FGA, FGB and SERPINC1, are acute phase proteins which regulate the host immune response to inflammation [52]. Thus, ORF3 may regulate the immune response to HEV infection by interacting with these proteins [25] (Ratra et al., 2008).

In summary, we identified 32 interactions between the HEV ORF3 protein and human proteins using a stringent, high throughput yeast two-hybrid system. Bioinformatic analysis showed that these interacting proteins have various cellular locations and are involved in many cellular processes, suggesting that the ORF3 protein is likely to have multiple biological functions. Analysis of the interaction network revealed enrichment of canonical pathways (p<0.05) encompassing complement and coagulation, cellular iron cation homeostasis and oxidative stress. A significant number of proteins interacting with the ORF3 protein are involved in multiple pathways associated with hemostasis. These results provide important guidance for further study of the pathogenesis of the ORF3 protein.

## Materials and Methods

### Yeast two-hybrid screening

Yeast two-hybrid assays were performed using Y2H Gold Matchmaker system (Clontech, Takara, USA) according to the manufacturer's protocols. Genotype 1 HEV ORF3 was used as bait against a human liver cDNA library for interaction screening by yeast mating. The coding region of ORF3 genotype 1 was amplified from recombinant plasmids containing cDNA of the W2-1 strain (GenBank Acc. JQ655734) [36]. The amplified ORF3 gene was then subcloned into the DNA-binding domain of Gal4 (Gal4-BD) in vector pGBKT7 to construct the bait plasmid, pGBKT7-HEV1ORF3. pGBKT7-HEV1ORF3 was then transformed into yeast strain Y2HGold (bait strain). Prior to yeast mating, bait plasmid was tested for auto-activation. A human liver cDNA library (Clontech, Takara, USA) was transferred into the pGADT7 vector, in which prey sequences are fused with the GLA4 transcription activation domain (AD) and expressed as a fusion protein of prey and AD. Each library was generated from random primed, directionally cloned cDNA, which was typically composed of over 1.3×10^8^ million independent clones with fragments ranging 0.5 to ≥3.0 kb.

Y189 yeast cells containing the liver cDNA plasmid were allowed to mate with Y2H cells by incubation at 28°C for 2 hours. The mating mixture was plated on synthetic medium lacking tryptophan, leucine, histidine, adenine and containing 150 ng/ml Aureobasidin A (AbA) (SD/-Trp/-Leu/X-a-gal/AbA medium) and then assayed for a-galactosidade activity. After 3–5 days culturing at 28°C, each of the colonies growing with a blue color was selected and plated on more stringent medium SD/-Ade/-His/-trp/-leu/X-α-Gal/AbA, which was also lacked histidine and adenine, for further selection. Plasmids were isolated from blue colonies growing on the stringent plate and used to transform E. coli DH5a cells. The prey constructs were rescued from E. coli cells, with selected using the appropriate antibiotic. The cDNA inserts of these plasmids were sequenced and the DNA sequences obtained were then used for a BLAST search against the GeneBank™ database.

The interactomes screened by yeast mating were furter confirmed by cotransformation of each pair of pGBKT7-HEV1ORF3 and prey constructs into Y2HGold. Each primary screening was carried out three times and all initial positive pairs from these three primary screens were co-transfected individually into Y2HGold twice for retesting for positive reactions. The final datasets contain those interaction pairs that were retested successfully twice without exhibiting autoactivation of the yeast reporter genes.

### Co-immunoprecipitation

Co-immonoprecipitation assays were performed using the “Matchmaker™ Chemiluminescent Co-IP System” (Clontech, Takara, USA) according to the manufacturer's instructions. In brief, the HEV ORF3 region was amplified using the bait plasmid pGBKT7-HEV1ORF3 as template and human liver cDNAs were amplified using pGADT7-prey constructs selected by two-hybrid screening as templates. The gene of HEV ORF3 cDNA was inserted into pAcGFP1 to construct the plasmid pAcGFP1-ORF3, which can expresses a GFP1 and ORF3 fusion protein. Each of the human liver cDNAs was inserted into vector pProLabel to generate in-frame ProLabel-prey fusion plasmids.

293FT cells (70 to 80% confluent) grown in 10-cm-diameter dishes in Dulbecco's modified Eagle medium (DMEM) supplemented with 10% fetal bovine serum were co-transfected with pAcGFP1-ORF3 plasmid encoding the HEV ORF3 and GFP1 fusion protein and pProLabel-prey plasmids encoding human and prolable-tag fusion proteins (0.8 µg of each plasmid DNA per dish). Transfection experiments were performed in Opti-MEM using Lipofectamine-Plus reagents (Invitrogen, Carlsbad, CA). Twenty four hours post transfection, the expression of AcGFP1-bait fusion protein was observed by fluorescent microscopy.

At 48 hr post-transfection, cells were pelleted and lysed in lysis buffer for co-immunoprecipitation analyses. The diluted lysate sample was incubated with either anti-HEV ORF3 polyclonal antibody or anti-AcGFP polyclonal antibody on a rotator at 4°C for 2 hr. And then the entire volume of each sample was transferred to a tube containing 25 ml of washed pre-clearing protein G plus/protein agarose beads. The tubes were rotated gently at 4°C overnight and the resulting immunocomplexes were precipitated on the agarose beads. The beads were then washed twice with whole cell extract buffer. The polyclonal antibody against ORF3 protein was made in our laboratory by immunizing rabbits with purified ORF3 protein expressed in E.coli.

ProLabel activity was detected using a ProLabel Detection Kit II (Clontech, Takara, USA). Three volumes of cell lysis buffer were combined with one volume of EA to prepare lysis/complementation buffer for each sample. For each ProLabel assay, 30 ml substrate were prepared by mixing 1.2 ml of Galacton, 6 ml of Emerald and 22.8 ml of Substrate. Each sample of beads was resuspended in 80 ml of the lysis/complementation buffer and then the entire content (beads and buffer) were transferred to a flat bottom well in a 96-well black plate. 30 ml of substrate mix was added and theproLabel signal intensity was read at 30 min after addition of substrate and expressed as relative luminescence units (RLU) of the samples using the GloMax® 96 Microplate Luminometer (Promega, USA).The pair GFP1-lam and Prolable-T provided by the manufacturer was as used as the negative control. Each interaction was tested independently three times and the average RLU was calculated. Statistical significance was based on an unpaired student's t-test.

### Construction and analysis of the interactome networks

All network graphical assemblies and manipulations were performed using Cytoscape 2.8 network visualization system [53]. Secondary interactors of ORF3 interacting proteins were retrieved from the BioGRID3.1 database linked to Cytoscape [54].

Gene ontology enrichment and pathway analysis was conducted with the web-based software Gene Set Enrichment Analysis (GSEA, version 3.0, Broad Institute http://www.broad.mit.edu/gsea). The gene sets used are from the Molecular Signatures Database (MsigDB) [55], catalog C2 functional sets, subcatalog canonical pathways, which include 880 gene sets from pathway databases (version 3.0). These gene sets are canonical representations of a biological process compiled by domain experts, BioCarta, Reactome and KEGG. Catalog C5 Go sets, GO associations retrieved from the GO consortium, GO gene sets are based on ontologies. We grouped the pathways derived directly from the GSEA into distinct pathway groups to avoid redundancies. The enrichment of specific biological associations within each network was estimated by Fisher's exact test (p≤0.05) using the module fisher calculated automatically.
